# Moxibustion Regulates the BRG1/Nrf2/HO-1 Pathway by Inhibiting MicroRNA-222-3p to Prevent Oxidative Stress in Intestinal Epithelial Cells in Ulcerative Colitis and Colitis-Associated Colorectal Cancer

**DOI:** 10.1155/2024/8273732

**Published:** 2024-09-25

**Authors:** Xuejun Wang, Haiyang Ji, Yanting Yang, Dan Zhang, Xiehe Kong, Xiaoying Li, Hongna Li, Yunqiong Lu, Guang Yang, Jie Liu, Huangan Wu, Jue Hong, Xiaopeng Ma

**Affiliations:** ^1^ Yueyang Hospital of Integrated Traditional Chinese and Western Medicine Shanghai University of Traditional Chinese Medicine, Shanghai 200437, China; ^2^ Eye Institute and Department of Ophthalmology Eye and ENT Hospital Fudan University, Shanghai 200030, China; ^3^ Shanghai Research Institute of Acupuncture and Meridian Shanghai University of Traditional Chinese Medicine, Shanghai 200030, China

## Abstract

Oxidative stress is crucial in ulcerative colitis (UC) and colitis-associated colorectal cancer (CAC). Intestinal epithelial cells (IECs) are an important component of the intestinal barrier. In previous studies, we have demonstrated that suppressing microRNA-222-3p (miR-222-3p) can protect against oxidative stress in IECs, which ameliorates colonic injuries in UC mice and prevents the conversion of UC to CAC. In this case, we hope to explore whether moxibustion can alleviate UC and CAC by inhibiting miR-222-3p based on mouse models of UC and CAC. After herb-partitioned moxibustion (HPM) intervention, the disease activity index (DAI) and colon macroscopic damage index (CMDI) were significantly reduced in UC mice, and the number and volume of intestinal tumors were decreased considerably in CAC mice. Meanwhile, we found that HPM suppressed miR-222-3p expression and upregulated the mRNA and protein expression of Brahma-related gene 1 (BRG1), nuclear factor erythroid 2-related factor 2 (Nrf2), heme oxygenase-1 (HO-1), while inhibiting Kelch-like ECH-associated protein 1 (Keap1) expression in IECs of UC and CAC mice. With changes in reactive oxygen species (ROS), malondialdehyde (MDA), glutathione peroxidase (GSH-Px), and inflammatory cytokines interleukin (IL)-1*β* and tumor necrosis factor (TNF)-*α*), we verified that HPM protects against oxidative stress and inflammation in IECs of UC and CAC mice. The effect of HPM was inhibited in miR-222-3p overexpression mice, further demonstrating that the protective effect of HPM on UC and CAC mice was through inhibiting miR-222-3p. In summary, HPM regulates the BRG1/Nrf2/HO-1 pathway by inhibiting miR-222-3p to attenuate oxidative stress in IECs in UC and CAC.

## 1. Introduction

Ulcerative colitis (UC) is a chronic inflammatory disease of the colonic mucosa that tends to develop between ages 30 and 40 [1, 2]. Epidemiological studies indicate that the annual incidence and prevalence rates of UC are between 1.2 and 20.3 per 100,000 and 7.6–245 per 100,000, respectively, and these rates are on an upward trajectory [1, 3]. Patients with UC show a higher risk of developing colorectal cancer (CRC) after a period of time, approximately 35 years [4], while sooner in North America and Asia [5]. It has been shown that the pathogenesis of UC is closely related to intestinal epithelial cells (IECs), since the damage of IECs leads to the destruction of the intestinal barrier, resulting in the luminal microflora triggering a sustained and uninhibited inflammatory response [6]. The chronic inflammatory response to UC and the abnormal proliferation of IECs will lead to the development of colitis-associated colorectal cancer (CAC) [7, 8, 9, 10].

Oxidative stress (OS) has long been recognized as a causal factor of UC and CAC [11]. Under physiological conditions, reactive oxygen species (ROS) in intestinal tissues have a bactericidal effect and are involved in intestinal defense functions [12]. However, OS arises from the overproduction of reactive oxygen species (ROS) exceeding the host's antioxidant defense capacity, can oxidize polyunsaturated fatty acids, triggering lipid peroxidation, which increases the permeability of cellular membranes, leading to intestinal mucosal barrier damage, bacterial translocation, and inflammatory responses [13]. The ROS generated by chronic inflammatory infiltration are thought to induce oxidative-based modifications in DNAs (i.e., 2-OH-cytosine, 8-oxo-adenine, and 8-oxo-guanine). These modifications may contribute to carcinogenesis through their potential for mispairing and mutagenesis [14, 15].

Noncoding RNAs known as microRNAs (miRNAs, miRs) are a conserved family that is named for their short size (18–22 nucleotides on average) [16]. miRNAs are found in almost all tissues and can control most (minimum 2/3) of protein-coding genes [17]. Thus, miRNAs are involved in various physiological and pathological processes by regulating target genes and target gene-related signaling [18, 19]. It has been found that upregulated microRNA-222-3p (miR-222-3p)- downregulates antioxidative genes via 3′UTR binding, meanwhile, upregulates pro-oxidative genes via TATA-box binding-mediated transcription activation, and it is the opposite for downregulated miR-222-3p [20, 21]. Our previous study has confirmed that inhibiting miR-222-3p can attenuate OS in IECs in UC and CAC [22].

Brahma-related gene 1 (BRG1), the central catalytic ATPase of the switch/sucrose nonfermentable (SWI/SNF) chromatin remodeling complex, alters the structure of chromatin in an ATP-dependent manner [23]. It was shown that BRG1 deficiency resulted in an excess of ROS in mouse colonic epithelial cells, generating OS and causing spontaneous enteritis or a high susceptibility to DSS-induced colitis and AOM/DSS-induced CAC [15, 24]. Under physiological conditions, as a central regulator that maintains intracellular redox homeostasis, nuclear factor erythroid 2-related factor 2 (Nrf2) is present in the cytoplasm and binds to Kelch-like ECH-associated protein 1 (Keap1) to form a complex that maintains an inhibitory state [25, 26, 27]. However, given OS, Nrf2 is released and translocated into the nucleus, activating antioxidant protein heme oxygenase-1 (HO-1) to exert antioxidant and anti-inflammatory effects [28].

Herb-partitioned moxibustion (HPM), a common form of moxibustion intervention, is an important component of external treatment in traditional Chinese medicine (TCM). A clinical study has confirmed the significant effect of moxibustion in improving the colitis activity index and inflammation in patients with mild-to-moderate active UC [29, 30]. Animal experiments have also partially revealed its mechanisms, such as modulating gut microbiome, repairing intestinal mucosa, and regulating abnormally expressed cytokines [31, 32, 33, 34, 35]. Moreover, moxibustion can also delay the progression of gastrointestinal tumors and CAC [36, 37].

**Figure 1:**
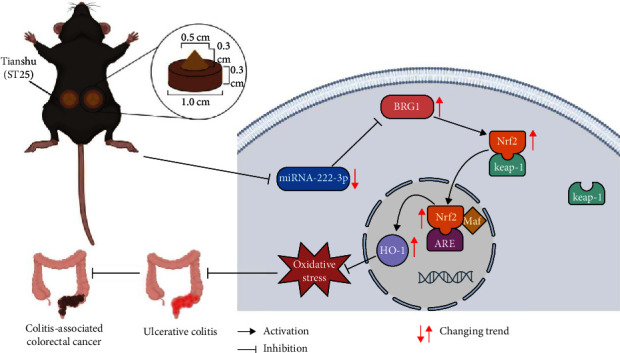
Illustration of the mechanism of HPM intervening UC and CAC. HPM leads to the inhibition of miR-222-3p to activate the BRG1/Nrf2/HO-1 pathway, protecting against oxidative stress and thereby relieving colonic inflammation in UC and inhibiting CAC. HPM, herb-partitioned moxibustion; UC, ulcerative colitis; CAC, colitis-associated colorectal cancer.

Our previous research has demonstrated that the inhibition of miR-222-3p can upregulate BRG1 and activate the Nrf2/HO-1 signaling pathway, thereby providing a defense against oxidative stress in IECs in UC and CAC [22]. However, the potential of HPM to alleviate oxidative stress in IECs in UC and CAC by inhibiting miR-222-3p remains unclear. Therefore, this study aims to seek whether HPM can activate the BRG1/Nrf2/HO-1 pathway by inhibiting miR-222-3p to counteract OS, thereby mitigating UC injuries and inhibiting the transition to CAC (Figure 1).

## 2. Materials and Methods

### 2.1. Experimental Animals

Male C57BL/6 mice (6–8 weeks old and weighing 20 ± 2 g) were obtained from Shanghai SLAC Laboratory Animal Co., Ltd. (Shanghai, China; License No: SYXK (Shanghai) 2018-0040). All animal experiment protocols were implemented in accordance with the International Guiding Principles for Biomedical Research Involving Animals recommended by the World Health Organization and were approved by the Ethics Committee of Yueyang Clinical Medicine School, Shanghai University of Traditional Chinese Medicine.

### 2.2. Grouping and Intervention

In the UC experiments, C57BL/6 mice were randomly divided into a normal control (NC) group, a UC group, an HPM group, and an AAV-222-3p overexpression plus HPM treatment (AOH) group. As a blank control, mice in the NC group were not intervened, and other mice were induced to be UC experimental models. Mice in the HPM group were treated with HPM after modeling, and in the AOH group, adeno-associated virus-green fluorescence protein vector 9 (AAV9-GFP) was injected via tail vein to induce overexpression of miR-222-3p 2 months before modeling, and other interventions were consistent with the HPM group.

In the CAC experiments, C57BL/6 mice were randomly divided into an NC group, a CAC group, a HPM group, and an AOH group. All mice other than those in the NC group will be induced as CAC models. In the HPM group, the HPM intervention was performed during modeling to verify the inhibition of HPM on the conversion of UC to CAC. Mice in the AOH group underwent the same intervention as in the HPM group 2 months after the tail vein injection of AAV9-GFP.

#### 2.2.1. Induction of UC and CAC

UC and CAC experimental models were prepared according to previous studies [15, 22]. In brief, an intraperitoneal injection of tamoxifen (100 mg/kg body weight) (Sigma–Aldrich, USA) for 3 consecutive days was performed to establish BRG1 deletion. The mice were fed 3% dextran sulfate sodium (DSS) (MW, 36–50 kDa; MP Biomedicals) for 3 days to induce UC models (Figure S1). In the CAC experiments, mice were intraperitoneally injected with AOM (10 mg/kg body weight) (Sigma–Aldrich, USA). After 7 days, 3% DSS was offered via drinking water for 4 days, followed by 16 days of normal drinking water; this treatment cycle was repeated three times (Figure S1). At the end of the modeling process, one mouse from each group was taken for pathological observation of the colon by hematoxylin and eosin (H&E) staining, and the next intervention was performed when the success of modeling was confirmed.

### 2.3. AAV9-GFP Construction and Tail Vein Injection

Based on previous studies [38], the miR-222-3p overexpression (AAV-222-3p overexpression) recombinant AAV9-GFP, obtained from Shanghai Genechem Co., Ltd. (Shanghai, China), was applied to transfect mice. In brief, 1 × 10^11^ particles of AAV in 200 *μ*L PBS were injected into the tail vein of mice. Two months later, the colon was harvested to measure the fluorescence intensity of AAV9-GFP with a fluorescence microscope. The successful transfection was identified by detecting the expression of miR-222-3 in IECs.

### 2.4. Herb-Partitioned Moxibustion

As shown in Figure 1, we ground the medicinal materials mainly composed of monkshood (a herb, the roots of Aconitum) into powder and added millet wine to make a round cake (1 cm in diameter and 0.3 cm in thickness) with a moxa cone (0.5 cm in diameter and 0.3 cm in thickness) placed on top. Bilateral Tianshu (ST25) points were chosen [39, 40]. For UC mice, HPM treatment was performed after modeling, with four moxa cones burned successively at each point as one session, once a day for a total of 7 days (Figure S1). For CAC mice, the HPM intervention was performed three times a week between days 11 and 71 of the modeling period, and four moxa cones were burned at each point each time (Figure S1).

### 2.5. Sample Collection and Processing

The symptoms of UC mice were evaluated by the disease activity index (DAI) and colon macroscopic damage index (CMDI). The DAI, involving body weight, stool consistency, and rectal bleeding, was measured according to a previous standard scoring system [41]. The scores are shown in Table S1. The mice were sacrificed, the abdominal cavity was opened, and the length and weight of the colon were recorded. Subsequently, the colon tissues were washed in ice-cold PBS to clear fecal residue for CMDI assessment [42]. The scores are shown in Table S2.

### 2.6. H&E Staining

After being fixed with 4% paraformaldehyde, colon tissues were embedded in paraffin and sliced into 4 *μ*m sections. The prepared paraffin sections were stained with H&E, sealed, and observed using a microscope (Olympus Corporation, Japan).

### 2.7. Isolation and Culture of IECs

IECs were isolated from mice and their purity was identified according to our previous study [21]. Briefly, colon tissue was rinsed, incubated, sieved, centrifuged, and then cultured overnight in an epithelial cell culture medium. The next day, IECs in the supernatant were collected after digestion with 0.25% trypsin (including EDTA). Then, the purity of IECs was determined by immunofluorescence (IF), observed with a fluorescence microscope, and photographed (Olympus Corporation, Japan) (Figure S2).

### 2.8. RNA Isolation and Quantitative Real-Time Polymerase Chain Reaction (qRT-PCR)

Total RNA was isolated from IECs with TRIzol reagent (Invitrogen, Carlsbad, CA, USA). The expression levels of miR-222-3p, BRG1, Nrf2, and HO-1 mRNAs were measured by qRT-PCR. For miRNA analysis, cDNA synthesis was carried out using an EZ-press microRNA reverse transcription kit (EZBioscience, USA). For mRNA analysis, total RNA was reverse transcribed using the PrimeScript RT reagent kit with a gDNA eraser (Takara, Japan). qRT-PCR was performed with the Roche Light Cycler 480 II using TB GreenTM Premix Ex TaqTM (Tli RNaseH Plus) (Takara, Japan). The relative amounts of transcripts were calculated by the 2^−*ΔΔ*^Ct method. To normalize the data, U6 served as the internal reference for miRNAs, and GAPDH was the internal reference for mRNAs. The specific primer sequences are shown in Table S3.

### 2.9. Western Blotting

The protein expression levels of BRG1, Nu-Nrf2, HO-1, Keap-1, and caspase-3 were detected by Western blotting. Generally, total proteins and nuclear proteins were extracted from IECs using RIPA buffer (Roche, Basel, Switzerland) and a nuclear protein extraction kit (Beyotime, Shanghai, China), respectively. Then, the total protein in the supernatant (40*μ*g/well) was separated by SDS-PAGE (10%) and transferred onto a PVDF membrane (Millipore, Boston, MA, USA). The membrane was blocked with 5% BSA and incubated with appropriate primary antibodies, including anti-BRG1 (Abcam, Cambridge, UK), anti-Nrf2 (CST, Danvers, MA, USA), anti-HO-1 (CST, Danvers, MA, USA), anti-Keap1 (Abcam, Cambridge, UK), anti-*β*-actin (Beyotime, Shanghai, China), and anti-Lamin B2 (CST, Danvers, MA, USA) at 4°C overnight. Afterward, the membrane was incubated with secondary antibodies (Beyotime, Shanghai, China) for 1 hr at room temperature. The proteins were detected by an enhanced chemiluminescence kit (Beyotime, Shanghai, China), and protein expression was analyzed using the Image J software.

### 2.10. Immunohistochemistry

Immunohistochemistry was performed to detect the expression of BRG1, Nrf2, and HO-1 in the colon tissues. Simply put, 4 *μ*m paraffin sections of colon tissue were deparaffinized by heating at 60°C, antigen retrieval was performed in citric acid, and endogenous peroxidase inactivation was performed using 3% hydrogen peroxide. After incubating anti-BRG1 antibody (1 : 200, Abcam, Cambridge, UK), anti-Nrf2 antibody (1 : 100, CST, Danvers, MA, USA), and anti-HO-1 antibody (1 : 100, CST, Danvers, MA, USA) overnight at 4°C, secondary antibodies (Beyotime, Shanghai, China) were added dropwise and color development was performed using a DAB (3,3′-diaminobenzidine) kit (Boster, Wuhan, China). The staining results were observed and photographed under a microscope (Olympus Corporation, Japan).

### 2.11. Determination of ROS

The 6-carboxy-2,7-dichlorodihydrofluorescein diacetate (DCFH-DA) ROS assay kit (Beyotime, Shanghai, China) was used to detect ROS generation in IECs. According to the instruction manual of the ROS assay kit, IECs were treated with DCFH-DA (10 mmol/l) and incubated for 20 min at 37°C. Then, the fluorescence intensity in IECs was detected using a fluorescence microplate reader (488 nm excitation wavelength and 525 nm emission wavelength).

### 2.12. Glutathione Peroxidase (GSH-Px) Assay and Malondialdehyde (MDA) Assay

According to the manufacturer's protocol, GSH-Px activities and MDA levels in IECs were determined using a total glutathione peroxidase assay kit (Beyotime, Shanghai, China) and a lipid peroxidation MDA assay kit (Beyotime, Shanghai, China), respectively.

### 2.13. Caspase-3 Activity Assay

According to the instruction manual of the caspase-3 activity assay kit (Beyotime, Shanghai, China), IECs were lysed with cell lysis buffer, and the supernatant was collected by centrifugation. Then, the supernatant was added to the assay buffer and caspase-3 substrate and incubated in 96-well plates (Ac-DEVD-pNA) at 37°C for 6 hr. The absorbance values at 405 nm were analyzed by a microplate reader (BioTek, USA).

### 2.14. Enzyme-Linked Immunosorbent Assay (ELISA)

IECs-free supernatants were collected, and the IL-1*β* and TNF-*α* levels were measured using IL-1*β* and TNF-*α* ELISA kits (Shanghai Simuwu Biotechnology Co., Ltd., Shanghai, China), respectively. The absorbance at 450 nm was measured by a microplate reader (BioTek, USA).

### 2.15. Statistical Analysis

All data were analyzed using SPSS 25.0 statistical software (IBM, Armonk, NY, USA). Except for the CMDI, all data were expressed as mean ± SD and analyzed using the one-way ANOVA test. The least significant difference (LSD) method was used when pairwise tests indicated that the variances of different groups were equal, and the Games–Howell method was used when the variances were unequal. CMDI data were expressed as the median (P25, P75) and were analyzed using a nonparametric test (Kruskal−Wallis). *P* < 0.05 was considered statistically significant.

## 3. Results

### 3.1. HPM Relieves Colonic Injuries in UC Mice

**Figure 2:**
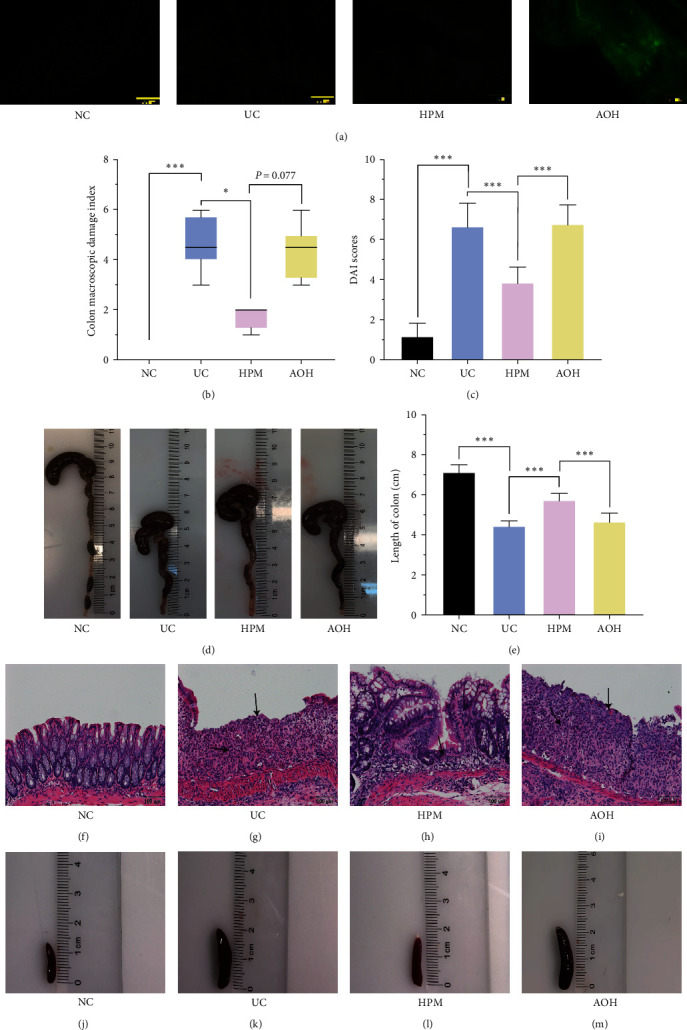
HPM relieves colonic injury and splenic enlargement. (a) Immunofluorescence analysis of frozen colon tissue sections showed that the AAV-222-3p overexpression agent was dramatically present in the colon in the AOH group. (b) Comparison of CMDI between different groups. (c) Comparison of DAI between different groups. (d) Comparison of colon images between different groups. (e) Comparison of colon length between different groups. (f–i) Morphological observation of hematoxylin and eosin (H&E)-stained colon sections. Arrows in (h) indicate ulcer healing sites, while in (g, i), they indicate ulcers and inflammatory cell infiltration sites. (j–m) Comparison of spleen images between different groups. Data are presented as the median (P25, P75) (*n* = 8) in (b) and mean ± SD in (c, e) (*n* = 8).  ^*∗*^*P* < 0.05,  ^*∗∗∗*^*P* < 0.001. NC, normal control; UC, ulcerative colitis; HPM, herb-partitioned moxibustion; AOH, AAV-222-3p overexpression plus herb-partitioned moxibustion.

To determine intestinal efficiency in vivo, frozen colon tissue sections were analyzed by fluorescence to measure the expression of the AAV-222-3p overexpression agent in the mice, and Figure 2(a) shows that the AAV-222-3p overexpression agent was dramatically present in the colon of mice in the AOH group.

Mice in the UC group exhibited significant appetite and weight loss, diarrhea, mucus bloody stool, and colon injuries and had higher DAI (*P* < 0.001) and CMDI (*P* < 0.001) than mice in the NC group (Figures 2(b) and 2(c)). The HPM intervention decreased DAI (*P* < 0.05) and CMDI (*P* < 0.001) compared with the UC group. However, the improvement effect of HPM on CMDI was significantly inhibited in the AOH group (*P* < 0.001) (Figures 2(b) and 2(c)). The shortening of the colon can show the severity of colon injury. Compared with the NC group, there was a decrease in colon length in the UC group and AOH group (*P* < 0.001); in contrast, compared with the UC and AOH groups, the colons of mice in the HPM group were considerably longer (*P* < 0.001) (Figures 2(d) and 2(e)).

The colonic structure was observed using H&E staining. Intestinal epithelial necrosis, goblet cell disorder and loss, continuous ulcers, and notable inflammatory cell infiltration in the mucosa and submucosa were observed in the UC group (Figure 2(g)). After HPM intervention, colonic injuries were attenuated, and damage to intestinal epithelial tissue, goblet cells, and glands was repaired, which shows that HPM ameliorates inflammatory damage in the colon of UC mice (Figure 2(h)). In the AOH group, dramatic ulcer formation reached the colonic mucosal layer, and a large number of inflammatory cells infiltrated the mucosal layer and submucosa (Figure 2(i)).

Meanwhile, we observed changes in spleen size between different groups. As shown in Figures 2(j), 2(k), 2(l), and 2(m), compared with the NC group, the spleen volume of mice in the UC group increased (Figure 2(k)). HPM intervention reduced splenic enlargement in UC mice (Figure 2(l)); however, mice in the AOH group still had a larger spleen (Figure 2(m)).

### 3.2. HPM Activates BRG1/Nrf2/HO-1 Pathway in UC Mice IECs by Inhibiting miR-222-3p

**Figure 3:**
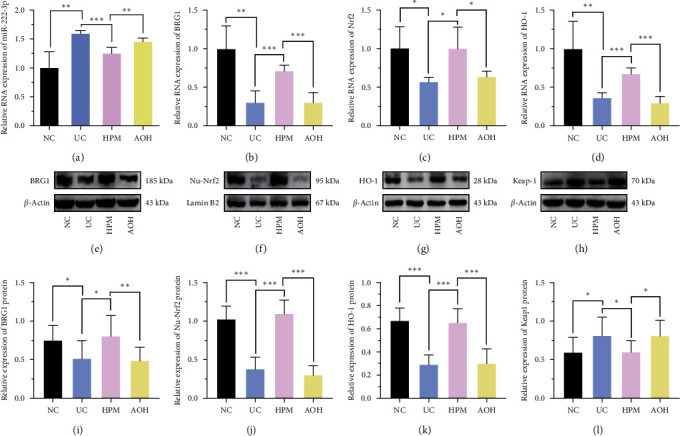
HPM activates the BRG1/Nrf2/HO-1 pathway in UC mice IECs by inhibiting miR-222-3p. (a) qRT-PCR analysis of miR-222-3p in the IECs from mice. (b–d) Relative BRG1, Nrf2, and HO-1mRNA expression levels were determined by qRT-PCR in the IECs from mice. Relative BRG1 (e, i), Nu-Nrf2 (f, j), HO-1 (g, k), and Keap1 (h, l) protein expression levels were determined by Western blot in the IECs from mice. Data are presented as the mean ± SD (*n* = 8).  ^*∗*^*P* < 0.05,  ^*∗∗*^*P* < 0.01,  ^*∗∗∗*^*P* < 0.001. NC, normal control; UC, ulcerative colitis; HPM, herb-partitioned moxibustion; AOH, AAV-222-3p overexpression plus herb-partitioned moxibustion.

To verify whether HPM ameliorates colonic injuries in UC mice through miR-222-3p, qRT-PCR was applied to detect the expression of miR-222-3p in each group of IECs. The miR-222-3p expression in the UC group was significantly higher than that in the NC group (*P* < 0.01). In the HPM group, the expression of miR-222-3p was reduced compared to the UC group (*P* < 0.001); however, the effect of HPM was abolished after the transfection with AAV-222-3p overexpression agent (*P* < 0.001), which also suggests successful transfection (Figure 3(a)).

Then, we proceeded to apply qRT-PCR to probe the levels of BRG1, Nrf2, and HO-1 mRNAs. As shown in Figures 3(b), 3(c), and 3(d), the levels of BRG1 (*P* < 0.01), Nrf2 (*P* < 0.05), and HO-1 (*P* < 0.01) mRNAs were decreased in the UC group compared with the NC group, while their levels were increased in the HPM group compared with the UC group (BRG1, *P* < 0.001; Nrf2, *P* < 0.05; HO-1, *P* < 0.001). Changes brought about by HPM were eliminated in the AOH group (BRG1, *P* < 0.001; Nrf2, *P* < 0.05; HO-1, *P* < 0.001).

Western blotting was performed to detect protein expression. After HPM intervention, the protein expression levels of BRG1 (*P* < 0.05), HO-1 (*P* < 0.001), and Nu-Nrf2 (*P* < 0.001) were significantly increased compared with the UC group, but this phenomenon was not observed in the AOH group (BRG1, *P* < 0.01; Nu-Nrf2, *P* < 0.001; HO-1, *P* < 0.001) (Figures 3(e), 3(f), 3(g), 3(i), 3(j), and 3(k)). Moreover, HPM decreased the protein expression of Keap1 (*P* < 0.05) (Figures 3(h) and 3(l)). The immunohistochemical findings further corroborate the impact of HPM intervention on the expression of BRG1, Nrf2, and HO-1(Figure S3). The above results suggest that HPM activates the BRG1/Nrf2/HO-1 pathway in IECs of UC mice by inhibiting the expression of miR-222-3p.

### 3.3. HPM Relieves OS and Inflammation in IECs of UC Mice

**Figure 4:**
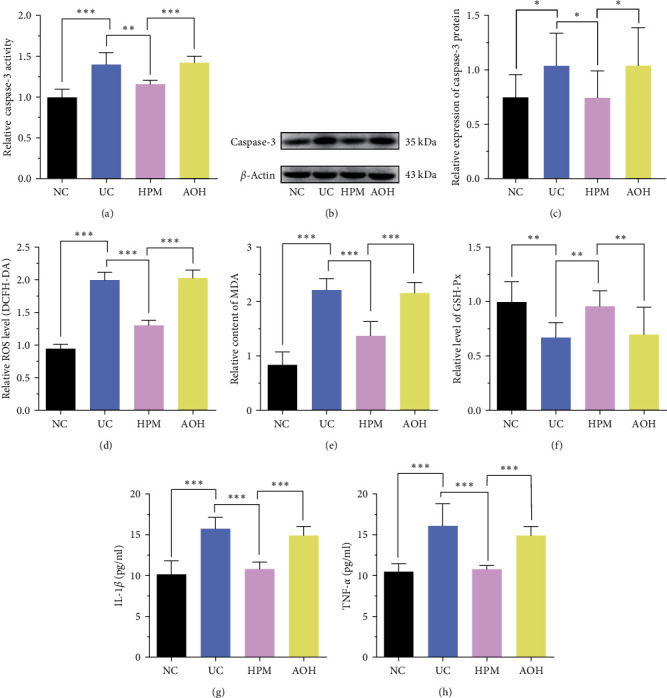
HPM relieves oxidative stress and inflammation in IECs of UC mice. (a) Comparison of the caspase-3 activity to assess IECs apoptosis. (b, c) Comparison of the relative caspase-3 protein expression. (d) Comparison of the ROS level measured by the DCFH-DA ROS assay kit. (e) Comparison of the MDA content. (f) Comparison of the GSH-Px level. (g, h) Comparison of the IL-1*β* and TNF-*α* expression levels determined by ELISA. Data are presented as the mean ± SD (*n* = 8).  ^*∗*^*P*  < 0.05,  ^*∗∗*^*P*  < 0.01,  ^*∗∗∗*^*P*  < 0.001. NC, normal control; UC, ulcerative colitis; HPM, herb-partitioned moxibustion; AOH, AAV-222-3p overexpression plus herb-partitioned moxibustion.

We further explored the effect of HPM on oxidative stress (OS) and inflammation in UC mice. The IECs of UC mice showed significant apoptosis (caspase-3 activity, *P* < 0.001; caspase-3 protein, *P* < 0.05), which was ameliorated by HPM intervention (caspase-3 activity, *P* < 0.01; caspase-3 protein, *P* < 0.05). In the AOH group, however, HPM did not reverse this apoptosis (caspase-3 activity, *P* < 0.001; caspase-3 protein, *P* < 0.05) (Figures 4(a), 4(b), and 4(c)). In addition, the ROS and MDA levels in IECs were distinctly increased after DSS treatment (ROS, *P* < 0.001; MDA, *P* < 0.001). HPM markedly reduced ROS and MDA levels in IECs of UC mice (ROS, *P* < 0.001; MDA, *P* < 0.001), but no changes in ROS and MDA levels were observed in the AOH group (ROS, *P* < 0.001; MDA, *P* < 0.001) (Figures 4(d) and 4(e)). Regarding the GSH-Px level in IECs, it was reduced after DSS treatment (*P* < 0.01) and returned to normal levels after HPM intervention (*P* < 0.01), yet remained significantly lower in the AOM group (*P* < 0.01) (Figure 4(f)).

To understand the situation further, we assessed the anti-inflammatory effect of HPM with changes in inflammatory cytokines. The ELISA results indicated that DSS increased the levels of IL-1*β* (*P* < 0.001) and TNF-*α* (*P* < 0.001) in the supernatant of IECs. After the HPM intervention, the levels of IL-1*β* (*P* < 0.001) and TNF-*α* (*P* < 0.001) were reduced, but in the AOH group, HPM did not achieve this effect (IL-1*β*, *P* < 0.001; TNF-*α*, *P* < 0.001) (Figures 4(g) and 4(h)).

These results suggest that HPM can improve apoptosis, oxidative stress, and inflammation levels in IECs of UC mice, and the activation of the BRG1/Nrf2/HO-1 pathway by inhibiting miR-222-3p is one way to achieve this effect.

### 3.4. HPM Prevents Intestinal Tumor Development in CAC Mice

**Figure 5:**
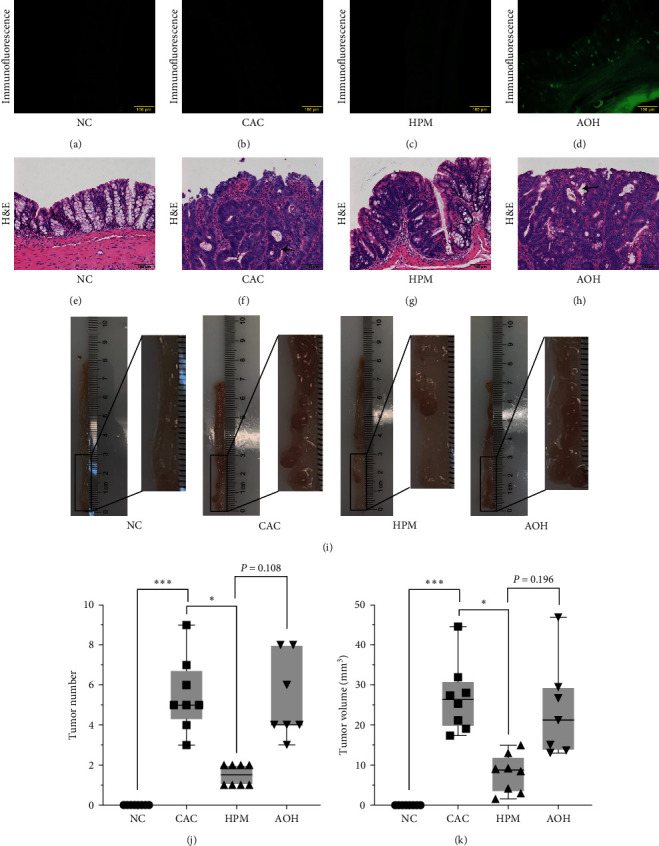
HPM prevents intestinal tumor development in CAC mice. (a–d) Immunofluorescence analysis of frozen colon tissue sections showed that the AAV-222-3p overexpression agent was dramatically present in the colon. (e–h) Morphological observation of H&E-stained colon sections. Arrows indicate large and deeply stained nuclei. (i) Macroscopic images of tumors in the intestines of mice. (j, k) Comparison of intestinal tumor number and volume. Data are presented as the median (P25, P75) in (d, e) (*n* = 7 in the AOH group, and *n* = 8 in the other groups).  ^*∗*^*P* < 0.05,  ^*∗∗∗*^*P* < 0.001. NC, normal control; CAC, colitis-associated colorectal cancer; HPM, herb-partitioned moxibustion; AOH, AAV-222-3p overexpression plus herb-partitioned moxibustion.

As immunofluorescence analysis of frozen colon tissue sections shows that the AAV-222-3p overexpression agent was dramatically present in the colon (Figure 5(d)). The colon images showed that mice with CAC developed more and larger tumors than normal controls (tumor number, *P* < 0.001; tumor volume, *P* < 0.001) (Figures 5(i), 5(j), and 5(k)). The number and volume of colonic tumors were significantly smaller in HPM mice compared with CAC mice (tumor number, *P* < 0.001; tumor volume, *P* < 0.001); however, we did not observe a reduction in the AOH group (tumor number, *P*=0.108; tumor volume, *P*=0.196) (Figures 5(i), 5(j), and 5(k)).

The histological analysis revealed that tumor cells could break through the basement membrane and grow invasively outward, and the nuclei became enlarged and hyperchromatic, reaching the level of adenocarcinoma in the CAC and AOH groups (Figures 5(e), 5(f), and 5(h)). In sharp contrast, HPM largely curtailed tumor development; the developed lesions were mainly graded as mild-to-moderate dysplasia, and slightly enlarged and hyperchromatic nuclei were observed (Figure 5(g)). This result suggests that HPM may inhibit intestinal tumors by suppressing miR-222-3p.

### 3.5. HPM Activates the BRG1/Nrf2/HO-1 Pathway by Inhibiting miR-222-3p in IECs to Prevent the Progression of CAC in Mice

**Figure 6:**
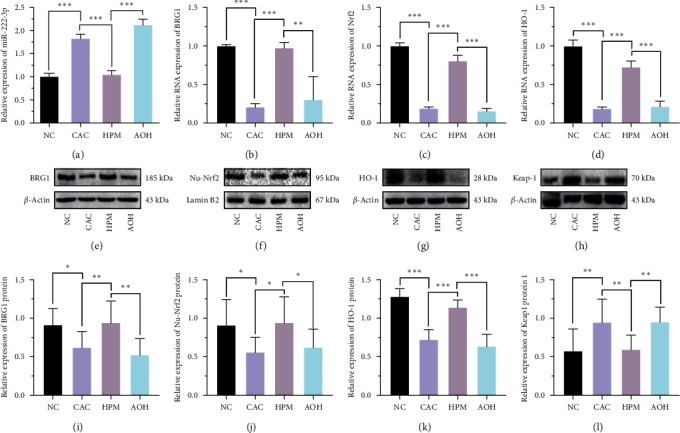
HPM activates the BRG1/Nrf2/HO-1 pathway by inhibiting miR-222-3p in CAC mice. (a) qRT-PCR analysis of miR-222-3 p in the IECs from mice. (b–d) Comparison of the relative expression of BRG1, Nrf2, and HO-1 mRNAs in IECs from mice. Comparison of the relative expression of BRG1 (e, i), Nu-Nrf2 (f, j), HO-1 (g, k), and Keap1 (h, l) proteins in IECs from mice. Data are presented as the mean ± SD (*n* = 7 in the AOH group and *n* = 8 in the other groups).  ^*∗*^*P* < 0.05,  ^*∗∗*^*P* < 0.01,  ^*∗∗∗*^*P* < 0.001. NC, normal control; CAC, colitis-associated colorectal cancer; HPM, herb-partitioned moxibustion; AOH, AAV-222-3p overexpression plus herb-partitioned moxibustion.

The qRT-PCR was used to detect the expression of miR-222-3p in IECs to confirm whether HPM inhibits intestinal tumors in CAC mice by suppressing miR-222-3p. The expression of miR-222-3p was significantly increased in IECs of CAC mice (*P* < 0.001), but it was reduced after HPM intervention (*P* < 0.001). Meanwhile, the inhibitory effect of HPM on miR-222-3p was blocked in the AOH group (*P* < 0.001) (Figure 6(a)).

According to the results in UC mice, qRT-PCR was used to detect the expression of BRG1, Nrf2, and HO-1 mRNAs in IECs of CAC mice. We found a significant decrease in BRG1, Nrf2, and HO-1 mRNAs in IECs of CAC mice (BRG1 mRNA, *P* < 0.001; Nrf2 mRNA, *P* < 0.001; HO-1 mRNA, *P* < 0.001). The HPM intervention increased BRG1, Nrf2, and HO-1 mRNAs in IECs (BRG1 mRNA, *P* < 0.001; Nrf2 mRNA, *P* < 0.001; HO-1 mRNA, *P* < 0.001), but the AAV-222-3p overexpression agent reversed this change (BRG1 mRNA, *P* < 0.01; Nrf2 mRNA, *P* < 0.001; HO-1 mRNA, *P* < 0.001) (Figures 6(b), 6(c), and 6(d)). We also performed Western blotting and immunohistochemistry to detect the protein expression of BRG1, Nu-Nrf2, and HO−1 in IECs, and the results were consistent with the mRNA results (Figures 6(e), 6(f), 6(g), 6(i), and 6(k)), Figure S4). Moreover, HPM decreased the expression of Keap1 (*P* < 0.01) (Figures 6(h) and 6(l)).

### 3.6. HPM Defends against OS and Inflammation by Inhibiting miR-222-3p in IECs of CAC Mice

**Figure 7:**
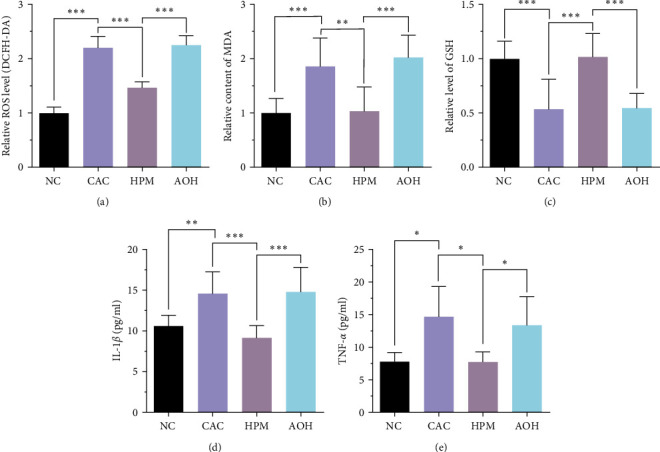
HPM protects IECs from oxidative stress and inflammation in CAC mice. (a) Comparison of the IECs ROS level measured by the DCFH-DA ROS assay kit. (b) Comparison of the MDA content. (c) Comparison of the GSH-Px level. (d–e) Comparison of the IL-1*β* and TNF-*α* levels. Data are presented as the mean ± SD.  ^*∗*^*P* < 0.05,  ^*∗∗*^*P* < 0.01,  ^*∗∗∗*^*P* < 0.001. NC, normal control; CAC, colitis-associated colorectal cancer; HPM, herb-partitioned moxibustion; AOH, AAV-222-3p overexpression plus herb-partitioned moxibustion.

We further extracted the supernatant of IECs to detect the OS and inflammation levels in CAC mice. In the CAC group, the levels of both ROS and MDA were significantly increased (ROS, *P* < 0.001; MDA, *P* < 0.001), but HPM intervention reduced the levels of ROS (*P* < 0.001) and MDA (*P* < 0.01) (Figures 7(a) and 7(b)). After transfection with AAV-222-3p overexpression agent, the effect of HPM was eliminated (ROS, *P* < 0.001; MDA, *P* < 0.001) (Figures 7(a) and 7(b)). Regarding GSH-Px, HPM intervention increased the level of GSH-Px in IECs of CAC mice (*P* < 0.001) but had no effect in the AOH group (*P* < 0.001) (Figure 7(c)).

Concerning the indicators of inflammation, IL-1*β* and TNF-*α* were significantly increased in the supernatant of IECs from CAC mice (IL-1*β*, *P* < 0.01; TNF-*α*, *P* < 0.05), but a decrease occurred after HPM (IL-1*β*, *P* < 0.001; TNF-*α*, *P* < 0.05) (Figures 7(d) and 7(e)). HPM did not exert its effect in the AOH group (IL-1*β*, *P* < 0.001; TNF-*α*, *P* < 0.05) (Figures 7(d) and 7(e)).

Considering that HPM intervention was performed for 60 consecutive days in the CAC mice, we performed H&E staining on the heart, liver, lung, spleen, and kidney tissues to evaluate whether HPM could cause adverse effects on mice. As shown in the results, compared with the NC group, significant damage was not observed in the HPM group or the AOH group (Figure S5).

With the results above, we conclude that HPM can inhibit CAC by suppressing miR-222-3p in the IECs and activating the BRG1/Nrf2/HO-1 pathway to protect against OS and inflammation.

## 4. Discussion

As a crucial component of TCM external treatment, moxibustion therapy has attracted more and more attention in recent years [43]. In previous studies, moxibustion has proven its positive effects on UC in a variety of ways, such as modulating gut microbiome, repairing intestinal mucosa, and regulating abnormally expressed cytokines [31, 32, 33, 44, 45], as well as inhibiting the development of gastrointestinal tumors and CAC [36, 37].

miRNAs are key regulators of the colonic epithelial barrier, regulating the growth and apoptosis of IECs and the tight junctions between IECs [46]. It has been shown that increased expression of miR-222-3p exacerbates the inflammatory response [47, 48, 49] and accelerates the progression of multiple tumor diseases by inhibiting apoptosis and promoting cancer cell proliferation and migration [50, 51, 52]. Our previous study [22] demonstrated that suppressing miR-222-3p is essential to alleviating UC and CAC by activating the BRG1/Nrf2/HO-1 pathway to protect IECs from OS. In this case, we want to know whether HPM activates the BRG1/Nrf2/HO-1 signaling by suppressing miR-222-3p expression, thus achieving a protective effect against UC and CAC.

There is evidence that miR-222-3p is highly expressed in the colonic tissues of UC and CAC patients [53, 54], which is consistent with our findings in UC and CAC mice (Figures 3(a) and 6(a)). In this study, UC and CAC experimental models were established and treated with HPM to observe whether HPM has a protective effect on UC and CAC. Meanwhile, we set up the AOH group to verify whether HPM acts on UC and CAC by suppressing miR-222-3p.

The results of qRT-PCR confirmed that HPM treatment reduces the level of miR-222-3p in IECs of UC and CAC mice (Figures 3(a) and6(a)). Meanwhile, we found that HPM intervention could alleviate the severity of colonic injury and reduce spleen volume in UC mice (Figures 2(b), 2(c), 2(d), 2(e), 2(f), 2(g), 2(h), 2(i), 2(j), 2(k), 2(l), and 2(m)), and HPM intervention, in the CAC experiment, reduced the number and volume of colonic tumors and attenuated heterotypic hyperplasia of the colonic epithelium (Figures 5(e), 5(f), 5(g), 5(i), 5(j), and 5(k)), which further confirms that the protective effect of HPM on UC and CAC is achieved by suppressing the expression of miR-222-3p.

OS, an essential role in the pathogenesis of UC and CAC [55], is closely related to miR-222-3p [20]. A previous study has shown that the ROS level is higher in the colon than in other parts of the body, which can lead to a more susceptible colon to damage [56]. When ROS exceeds the buffering capacity of antioxidant defenses, it leads to lipid peroxidation, which damages epithelial cells and leads to disruption of the intestinal mucosal barrier, resulting in local chronic inflammation [13, 57]. Simultaneously, OS causes DNA damage and atypical epithelial cell proliferation, which may eventually lead to CAC [58]. Therefore, protecting colonic epithelial cells from damage by OS is considered significant for UC and CAC. Our results proved that the ROS level was higher in IECs of UC and CAC mice, but then decreased after HPM intervention (Figures 4(d) and 7(a)). Meanwhile, by measuring GSH-Px and MDA, important markers of OS, we further confirmed that HPM could resist OS to alleviate UC and CAC by inhibiting miR-222-3p (Figures 4(e), 4(f), 7(b), and 7(c)).

According to previous research, BRG1 maintains the homeostasis of IECs to prevent intestinal inflammation and tumorigenesis [15]. Defective autophagy in BRG1-deficient IECs leads to an excess of ROS, which results in defective barrier integrity [15]. Not only associated with the growth of CRC [59], BRG1 is also critical in the metastasis and prognosis [60, 61, 62]. Activating the Nrf2/HO-1 pathway to resist OS and alleviate inflammation has proven to be an effective method for UC [63, 64], in which the suppression of ferroptosis may also play a part [65, 66]. Moreover, mitochondrial DNA damage is also alleviated following the reduction of OS, which also plays an essential role in preventing and controlling CRC [67]. Therefore, upregulation of BRG1 expression to activate the Nrf2/HO-1 signaling pathway is an important way to protect from UC and CAC [68, 69].

In this study, we verified that HPM upregulated BRG1, Nrf2, and HO-1 mRNAs in IECs of UC and CAC mice and increased the protein expression of BRG1, Nu-Nrf2, and HO-1, while inhibiting Keap1 protein expression. However, this effect was reversed after transfection with AAV-222-3p (Figures 3(b), 3(c), 3(d), 3(e), 3(f), 3(g), 3(h), 3(i), 3(j), 3(k), 3(l), 6(b), 6(c), 6(d), 6(e), 6(f), 6(g), 6(h), 6(i), 6(j), 6(k), and 6(l)). These results demonstrate that HPM activates the BRG1/Nrf2/HO-1 pathway by inhibiting miR-222-3p.

With changes in the caspase-3 level, we found that HPM inhibits the apoptosis of IECs in UC mice (Figures 4(a), 4(b), and 4(c)). In terms of inflammatory factors, changes in TNF-*α* and IL-1*β* levels show that HPM treatment can significantly improve colonic inflammation in UC and CAC mice, and this effect should be achieved by suppressing miR-222-3p (Figures 4(g), 4(h), 7(d), and 7(e)).

Furthermore, in the H&E staining of different organs of mice in the CAC experiment, we did not find any significant organ damage caused by HPM (Figure S3). Indeed, to evaluate the safety of HPM more accurately, more targeted studies should be conducted in the future.

In conclusion, we demonstrate that HPM can improve colonic injuries caused by OS and inflammation in UC mice, which is achieved by inhibiting miR-222-3p to activate the BRG1/Nrf2/HO-1 pathway; in addition, HPM protects against the damage of colonic epithelium to prevent the progression of UC to CAC. The current therapeutic strategies for UC predominantly rely on pharmacological interventions, such as 5-aminosalicylic acid, corticosteroids, and immunosuppressive agents [70]. In severe cases, colectomy remains a necessary procedure [71]. However, for patients with CAC, surgical intervention continues to be the primary mode of treatment [72]. Our study has provided a robust theoretical foundation for the clinical treatment of UC and CAC. Looking ahead, we envisage a collaborative endeavor with clinicians, public health experts, and statisticians to undertake clinical research on HPM for UC and CAC. These investigations will yield profound and valuable scientific insights, thereby optimizing treatment strategies and enhancing clinical practice.

Nevertheless, there are still some limitations in our study. Based only on a mouse model, we proved HPM upregulates BRG1/Nrf2/HO-1 by inhibiting miR-22-3p in UC and CAC, which has not been validated in humans. Additionally, we have scaled down the volume of the moxa cone (0.5 cm in diameter and 0.3 cm in thickness) based on the body weight of mice. However, in clinical practice, it appears that we may not be able to administer a treatment to patients with moxa cones of a proportionally equivalent size, which could be a critical aspect that warrants attention in future clinical studies. Moreover, when HPM exerts its antioxidant stress effects, the BRG1/Nrf2/HO-1 pathway may only be one of the routes. We have not explored other signaling pathways and thus cannot provide a more comprehensive explanation of the antioxidant mechanism of HPM. Further, the mechanism of HPM in treating UC and CAC may be multipathway, and miR-222-3p may only be one link. Also, multidisciplinary cross-sectional research using genomics and proteomics technologies is expected to study the mechanism of HPM in intervening UC and CAC.

## 5. Conclusions

In conclusion, HPM regulates the BRG1/Nrf2/HO-1 pathway by inhibiting miR-222-3p to attenuate oxidative stress in IECs in UC and CAC, and also, our experimental results will provide evidence support for the clinical application of HPM for UC and CAC.

## Data Availability

The original contributions presented in the study are included in the article/Supplementary Materials. Further inquiries can be directed to the corresponding authors.
