# Supplementary material for: Moxibustion Regulates the BRG1/Nrf2/HO-1 Pathway by Inhibiting MicroRNA-222-3p to Prevent Oxidative Stress in Intestinal Epithelial Cells in Ulcerative Colitis and Colitis-Associated Colorectal Cancer
Source: J Immunol Res. 2024 Sep 25;2024:8273732. doi: 10.1155/2024/8273732 (PMC11446618; doi:10.1155/2024/8273732)
Supplement: Supplementary Materials — The supplementary material for this article can be found online. [file 8273732.f1.doc]

**Supplementary Materials**

Supplementary Table 1 DAI Scoring

| The disease activity index | | | Score | |
| --- | --- | --- | --- | --- |
| Weight loss | | No loss | 0 | |
|  | | 5–10% | 1 | |
|  | | 10–15% | 2 | |
|  | | 15–20% | 3 | |
|  | | >20% | 4 | |
| Rectal bleeding | | No bleeding | 0 | |
|  | | occult blood 1+ | 1 | |
|  | | occult blood 2+ | 2 | |
|  | | occult blood 3+ | 3 | |
|  | | occult blood 4+ | 4 | |
| The appearance of diarrhea | | Absent | 0 | |
|  | | Mild diarrhea | 2 | |
|  | | Severe diarrhea | 4 | |
| Supplementary Table 2 CMDI Scoring | |  |  | |
| Colon macroscopic damage index | | | Score | |
| Colon adhesion | No adhesions | | | 0 |
|  | Minor adhesions | | | 1 |
|  | Major adhesions | | | 2 |
| Ulcer and inflammation | No ulcer or inflammation | | | 0 |
|  | Local congestion without ulcers | | | 1 |
|  | 1 ulcer without congestion or bowel wall thickening | | | 2 |
|  | 1 ulcer with inflammation | | | 3 |
|  | 2 ulcers with inflammation | | | 4 |
|  | > 2 ulcers with inflammation or inflammation area > 1 cm | | | 5 |
|  | Ulcer and inflammation area > 2cm; with one more damage, plus 1 point | | | 6~10 |

Supplementary Table 3 primer sequences

| Gene | Primer F | Primer R |
| --- | --- | --- |
| miR-222-3p | 5' AACACGCAGCTACATCTGGCTA3' | - |
| BRG1 | 5' GGTTCTGCCCACAGCATGAT3' | 5' GGACTCCATAGGCTTGTGCAT3' |
| Nrf2 | 5' CTTTAGTCAGCGACAGAAGGAC3' | 5' AGGCATCTTGTTTGGGAATGTG3' |
| HO-1 | 5' CAGAACCCAGTCTATGCCCC3 | 5' GTGAGGCCCATACCAGAAGG3' |
| GAPDH | 5' AGGTCGGTGTGAACGGATTTG3' | 5' TGTAGACCATGTAGTTGAGGTCA3' |
| U6 | 5' CCTGCTTCGGCAGCACA3' |  |

Supplementary Fig 1 Illustration of the modeling and intervention in UC and CAC

*
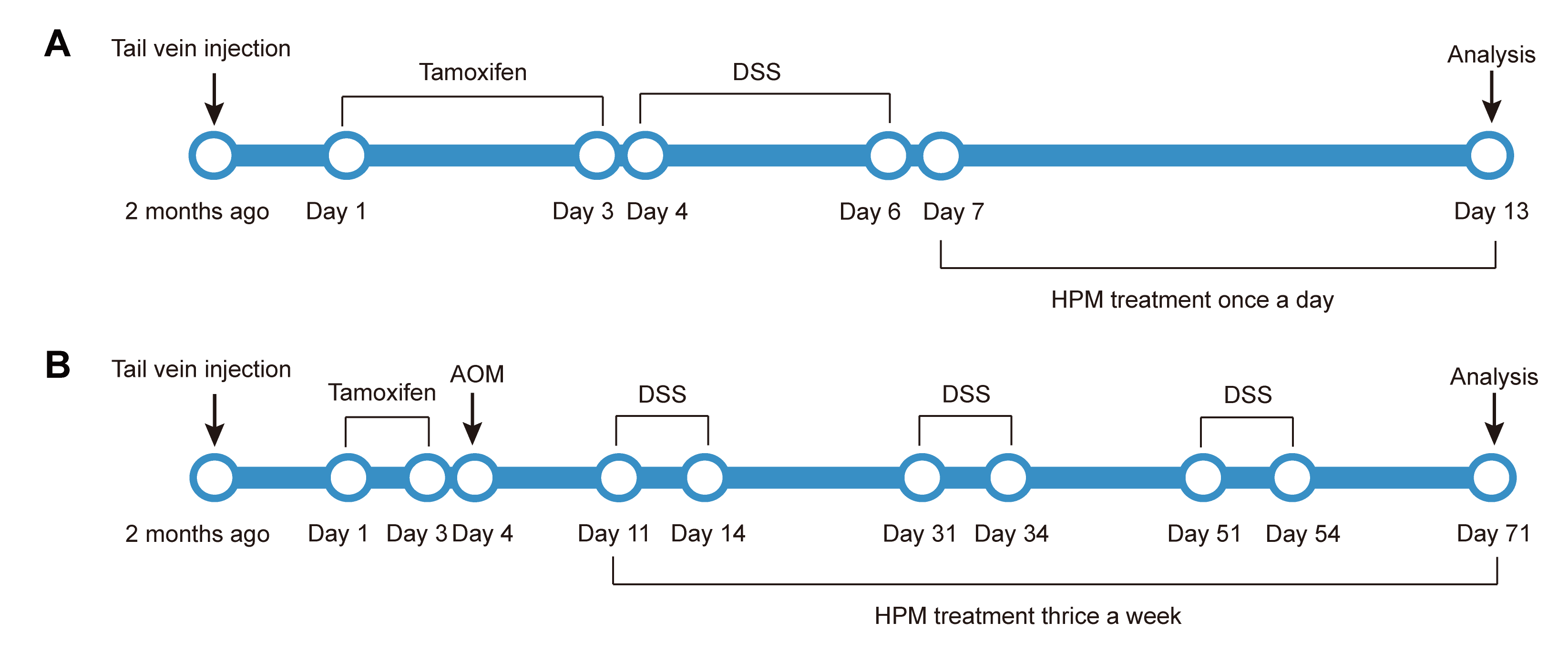
*

Supplementary Fig 1 (a) Timeline for modeling process and HPM intervention in UC mice. (b) Timeline for modeling process and HPM intervention in CAC mice. AOM: Azoxymethane; DSS: Dextran sulfate sodium.

Supplementary Fig 2 Fluorescence identification IECs


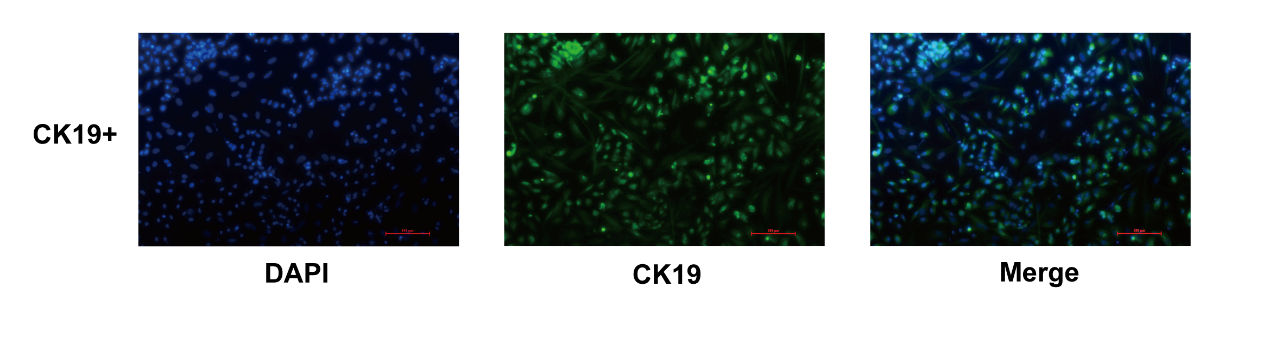


Supplementary Fig 2 Single immunofluorescent staining for cytokeratin 19 (CK 19+) was performed in IECs. Nuclei are stained with DAPI in blue. CK 19 is stained in green.

Supplementary Fig 3 HPM activates the BRG1/Nrf2/HO-1 pathway in UC mice


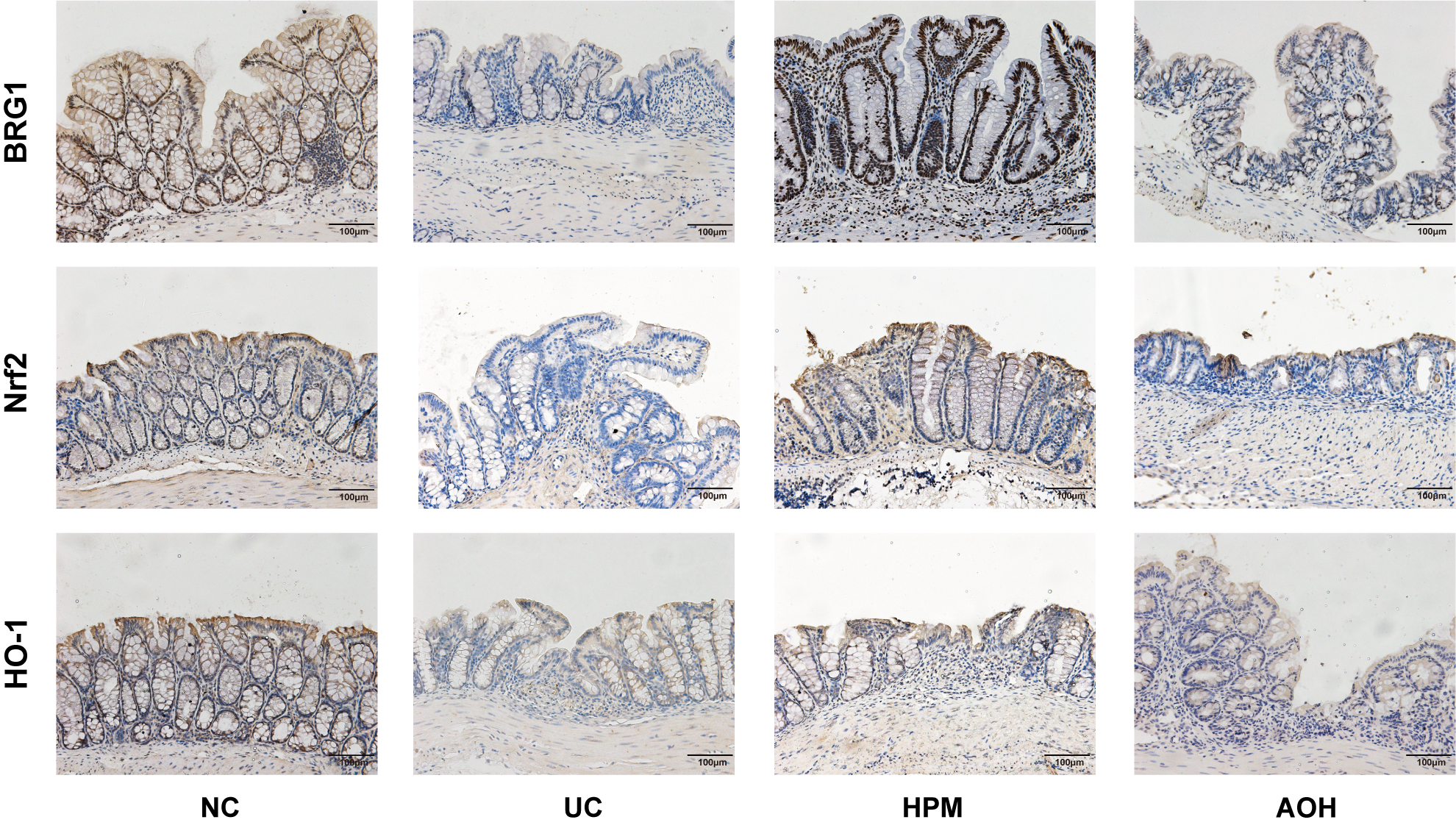


Supplementary Fig 3 Immunohistochemical staining of BRG1, Nrf2, and HO-1 of mice colon in UC experiments. NC: Normal control; UC: Ulcerative colitis; HPM: Herb-partitioned moxibustion; AOH: AAV-222-3p overexpression and herb-partitioned moxibustion treatment.

Supplementary Fig 4 HPM activates the BRG1/Nrf2/HO-1 pathway in CAC mice


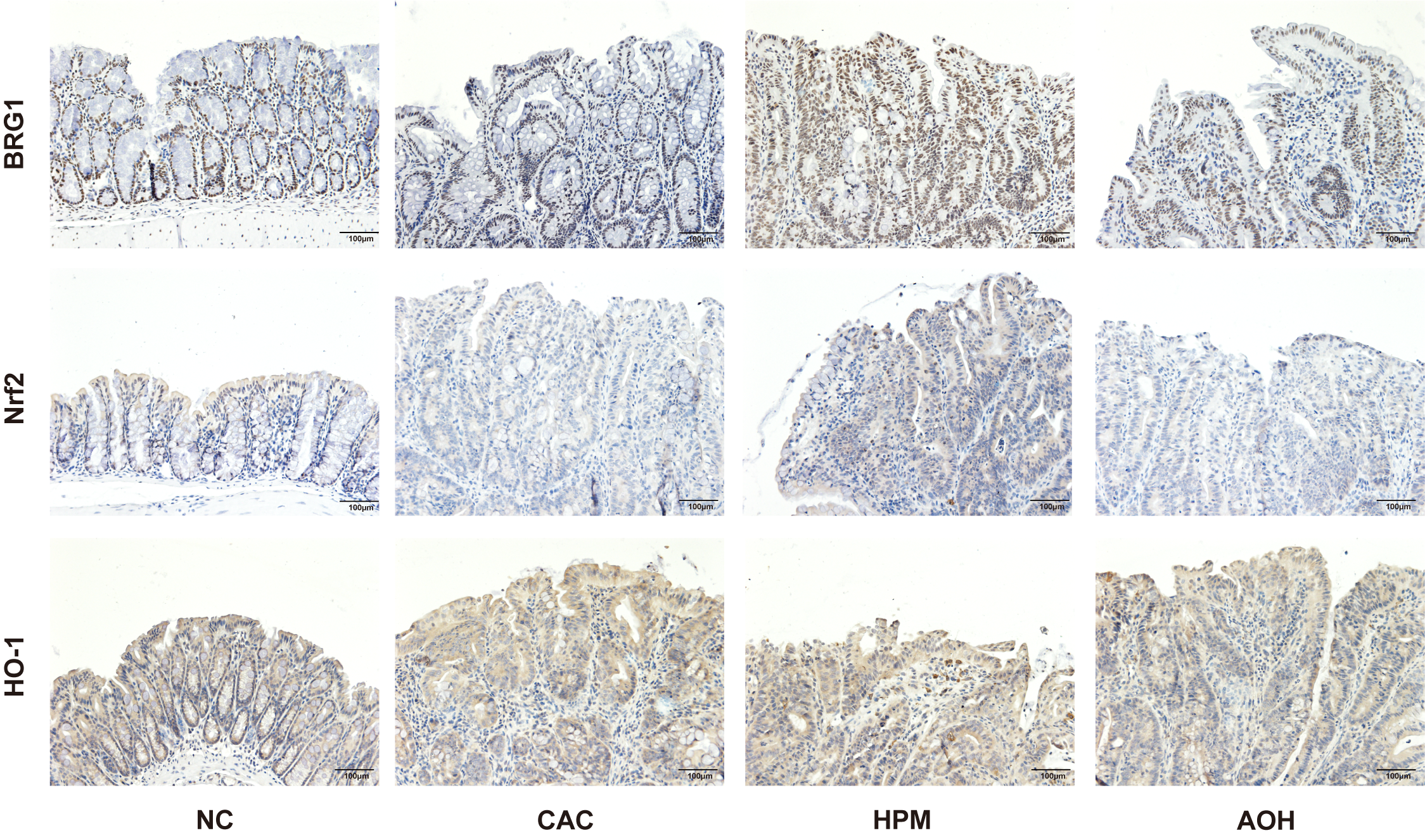


Supplementary Fig 4 Immunohistochemical staining of BRG1, Nrf2, and HO-1 of mice colon in CAC experiments. NC: Normal control; CAC: Colitis-associated colorectal cancer; HPM: Herb-partitioned moxibustion; AOH: AAV-222-3p overexpression and herb-partitioned moxibustion treatment.

Supplementary Fig 5 H＆E staining of different organs


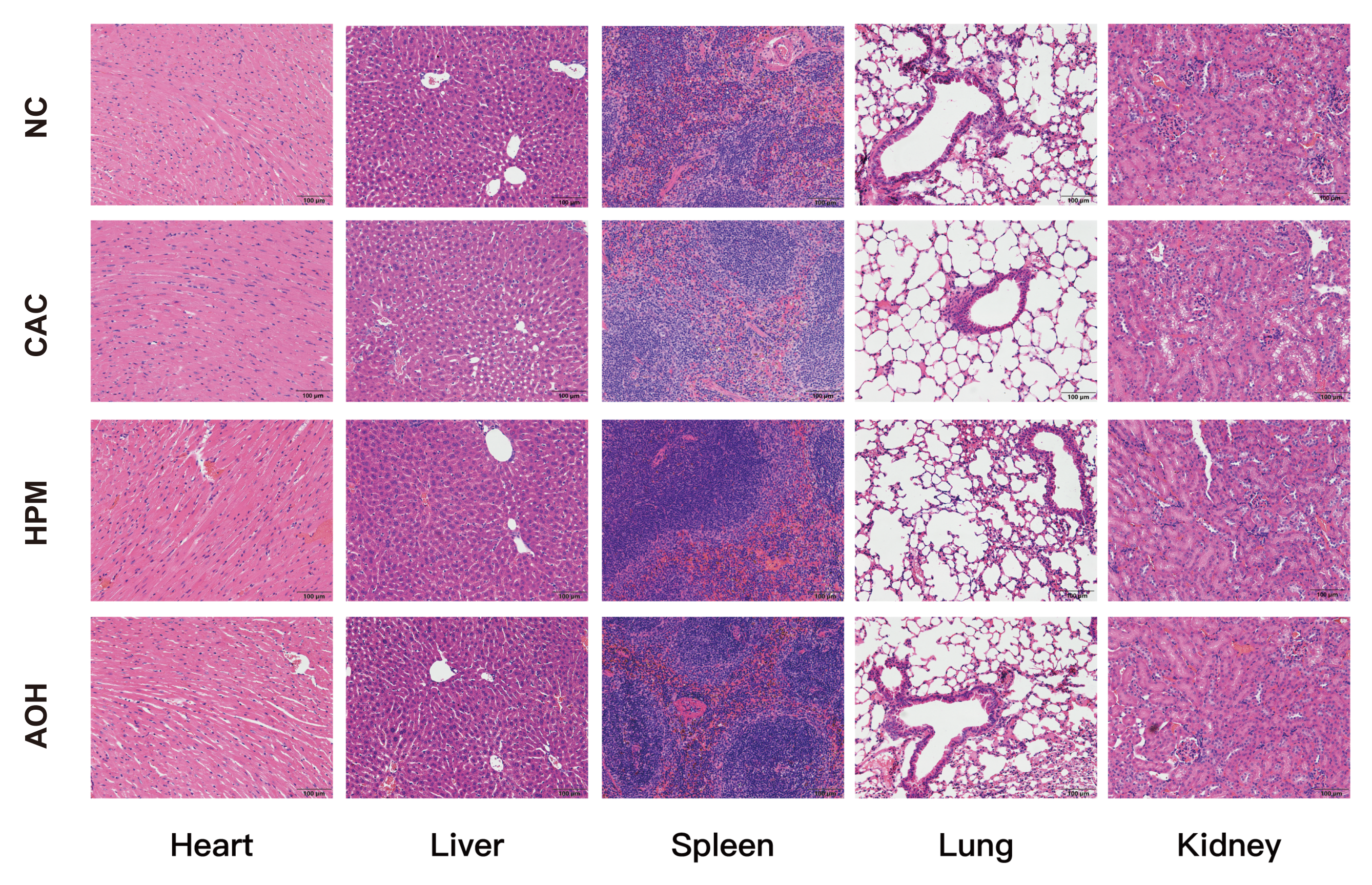


Supplementary Fig 5 H＆E staining of heart, liver, spleen, lung, and kidney of mice in the CAC experiment. NC: Normal control; CAC: Colitis-associated colorectal cancer; HPM: Herb-partitioned moxibustion; AOH: AAV-222-3p overexpression and herb-partitioned moxibustion treatment.
